# Talking it better: conversations and normative complexity in healthcare improvement

**DOI:** 10.1136/medhum-2020-012129

**Published:** 2021-05-25

**Authors:** Alan Cribb, Vikki Entwistle, Polly Mitchell

**Affiliations:** 1 Centre for Public Policy Research, King's College London, London, UK; 2 Health Services Research Unit and School of Divinity, History and Philosophy, University of Aberdeen, Aberdeen, UK

**Keywords:** inter-professional education, health policy, philosophy of medicine/health care, social science, medical ethics/bioethics

## Abstract

In this paper, we consider the role of conversations in contributing to healthcare quality improvement. More specifically, we suggest that conversations can be important in responding to what we call ‘normative complexity’. As well as reflecting on the value of conversations, the aim is to introduce the dimension of normative complexity as something that requires theoretical and practical attention alongside the more recognised challenges of complex systems, which we label, for short, as ‘explanatory complexity’. In brief, normative complexity relates to the inherent difficulty of deciding what kinds of changes are ‘improvements’ or, more broadly, what is valuable in healthcare. We suggest that explanatory and normative complexity intersect and that anyone interested in healthcare improvement needs to be sensitive to both. After briefly introducing the idea of normative complexity, we consider some contrasting examples of conversations, reflecting on how they do and might contribute to healthcare quality. We discuss both conversations that are deliberately organised and facilitated (‘orchestrated conversations’) and more informally occurring and routine conversations. In the first half of the paper, we draw on some examples of orchestrated and routine conversations to open up these issues. In the second half of the paper, we bring some more theoretical lenses to bear on both conversations and normative complexity, summarise what we take to be the value of conversations and draw together some of the implications of our discussion. In summary, we argue that conversations can play a crucial role in negotiating the normative complexity of healthcare quality improvement because of their capacity to hold together a plurality of perspectives, to contribute and respond to emergence and to help underpin institutional conditions for empathy and imagination.

Researchers with an interest in healthcare quality improvement, like those in many other fields, have begun to grapple with the challenges of complexity. We hope to make a contribution to that effort by highlighting the normative dimension of complexity in healthcare improvement. We are interested in exploring the role of conversations in responding to normative complexity, although for much of the paper the idea of normative complexity will remain largely in the background. After briefly introducing the idea, we consider some contrasting examples of conversations, reflecting on how they do and might contribute to healthcare quality. We will look at both what we call ‘orchestrated’ conversations and more routine conversations. In the remaining sections of the paper, we return more overtly to normative complexity, summarise what we take to be the value of conversations and draw together some of the implications of our discussion. In summary, what we will suggest is that conversations can play a crucial role in negotiating normative complexity because of their capacity to hold together a plurality of perspectives, to contribute and respond to emergence and to help underpin institutional conditions for empathy and imagination.

## Explanatory and normative complexity

Influential figures in healthcare quality improvement have underlined the importance of recognising that healthcare takes place within complex systems (Braithwaite 2018; Hollnagel 2014; Greenhalgh and Papoutsi 2018). Such work typically introduces ideas about complexity to shake up assumptions about causality. In particular, we are encouraged not to treat healthcare like a simple machine with clear lines of causality that ‘top-down’ improvers can straightforwardly translate into levers for change. Instead processes of causation, we are reminded, should be seen as non-linear and arising from interacting subsystems that are themselves evolving and parts of other ecosystems. These insights help to show why well-intentioned interventions based on reductionist ‘machine-like’ models will often fail. This is both because they do not deploy a wide enough angle of attention and, at the same time, because they do not get close enough to practice cultures and contexts to respond to the myriad of processes that sustain them. These concerns about ‘explanatory complexity’ to some extent overlap with, and have gained ground at the same time as, concerns about what we are calling ‘normative complexity’. In plain terms, what we are highlighting by using this term is that it is not just ‘the way things happen’ in healthcare that is complex, but also ‘what matters’—that is, what is, or should be taken as, valuable or successful in healthcare.

When deciding what to do in pursuit of healthcare improvement, we can be tripped up by either (or both) explanatory or normative complexity. The former should induce caution when making claims about the effects of our actions, the latter should induce caution when making claims about which such effects are improvements or, more broadly, about what counts as good and who ought to do what. We will not say much about it here (expanding on the subject in the penultimate section of the paper), but it is worth noting that there are resonances and intersections between explanatory and normative aspects of complexity. Separately and in combination, explanatory and normative complexity should lead to a sense of humility. They both entail that we need to think about relevant considerations not in isolation from one another but in ‘constellations’, including constellations formed at their intersection.

Unlike explanatory complexity, normative complexity has not featured explicitly in healthcare quality or improvement scholarship. However, at least one aspect of it features widely, although often implicitly, in quite routine, practice-oriented quality discourses. This is because there is now a general acceptance of the idea that for healthcare to be good it must respond to diverse value perspectives. Healthcare improvement approaches and practices increasingly take it for granted that biomedically defined outcomes are only one criterion of success, and that these need to be used alongside (or interpreted through the lens of) other conceptions of what matters to a range of ‘stakeholders’ (Cribb 2017; Entwistle et al. 2012; Ziebland et al. 2013). Most obviously, this means that professional perspectives on what is good need to be complemented with the perspectives of patients and informal carers. This complicates thinking about quality substantially including, for example, raising questions about how best and how far to incorporate and be guided by the values of specific groups and individuals—the latter being a prominent concern embodied in the language of person-centredness or, with somewhat different associations, personalisation. Normative complexity is also produced by the widespread recognition that ‘quality’ is multidimensional (eg, encompassing equity as well as person-centredness and safety as well as effectiveness (Institute of Medicine 2001)), and by complications arising from the growing attention to more diffuse healthcare agency encompassed in ideas such as partnership, shared decision-making and ‘asset-based’ working (Cribb and Entwistle 2011; Palmer, Weavell, and Callander 2019; Mitchell, Cribb, and Entwistle 2019). Such ideas raise questions about the division of labour and responsibility in healthcare—who can be, and should be, encouraged to be or held responsible for healthcare. This combination of diverse perspectives, multidimensionality and contestation about responsibility produces a level of complexity which is not easy to resolve; indeed, which is arguably inherently irresolvable.

Our working assumptions in what follows are: first, that a minimum condition of responding to normative complexity is creating systems and climates that do not bury or disguise its existence. Second, that various kinds of conversations have an important potential role in bringing to the surface, sometimes highlighting and sometimes actively addressing, aspects of normative complexity. We should stress that we are not proposing conversations as some kind of all purpose ‘solution’ for the complexities of healthcare quality improvement. Indeed, we hope to indicate some constraints and limitations as we go along. But we do think that attention to conversational possibilities can be one useful starting point for thinking about normative complexity.

In the second half of the paper, we will explore the idea of a ‘conversation’ in a little more depth—indicating some of the theoretical sources that inform our use of the term. But, in the main, we are relying on an everyday and relatively loose notion—where a conversation indicates a reciprocal communicative exchange between two or more people, often relatively informal in nature, through which information and perspectives are shared and, at the same time, relationships are built or sustained. As well as occurring fairly spontaneously, we take it that conversations can also be more or less orchestrated, and, in addition, more or less ‘directed’—the associations with informality stemming from the relatively unorchestrated and non-directed end of the spectrum. We are calling a conversation ‘orchestrated’ to the extent that it is deliberately organised and facilitated, and ‘directed’ to the extent that it exists, and is being shaped and used, to serve a specific institutional or professional purpose; that is, is deliberately oriented in a particular direction. The more directed such interpersonal exchanges are, the less the language of conversation seems to be a comfortable fit.

## Orchestrating conversations for better healthcare

We are interested in the potential contribution of the full range of conversations to improving healthcare but will begin with some reflections on a couple of high-profile examples of practices that are often overtly linked to healthcare quality improvement and which involve orchestrated conversations—Experience-Based Co-Design (EBCD) and Schwartz Rounds. Both arguably represent significant philosophical as well as practical interventions in healthcare, and we will draw attention to some contrasts between them. We also, however, indicate some of things they share in common.

EBCD is one of the more established, and hence theorised and researched, contributions to the now familiar ‘participatory zeitgeist’ of co-production (Palmer, Weavell, and Callander 2019). Its crucial component is the ‘bringing together’ of the experiences and voices of staff, patients and carers to work on healthcare improvement (Donetto et al. 2015). It is a carefully orchestrated process, which includes bringing different actors together both to share experiences and to explore, identify and pursue shared priorities. It is also a process that is ‘directed’ towards a particular end, namely service re-design and improvement. In other words, it uses conversational exchanges in a practical, ‘problem-solving’, way. Yet at the same time, it can be seen as a philosophical intervention because it incorporates the lived experiences of service-users in conversation with institutional actors thereby, among other things, helping to expand the agendas, horizons and working relationships of staff (and patients) and increasing the likelihood that services will be responsive to a broader range of concerns and values.

Schwartz Rounds are another influential and widely admired innovation in healthcare. They are a regular (often monthly) forum to which all members of staff (clinical and non-clinical) within a healthcare institution are invited in order to reflect on and discuss social, emotional and ethical challenges (Goodrich 2012). It would thus not be unreasonable to suggest that, in addition to their overt aims, they are expressly constructed so as to acknowledge and respond to what we are labelling as normative complexity. The rounds have broad ranging aims relating to staff well-being, engagement and learning and so, more indirectly, help underpin the quality of patient care. The meetings are carefully organised and facilitated by people trained in Schwartz principles, but the exchanges are not directed towards a specific institutional agenda. In practice, this means that there is no particular value attached towards agreement because there is no pressure towards the forms of convergence that are required for action planning. A meeting is no less successful for producing overlapping and untidy patterns of agreement, disagreement and with much remaining unresolved.

Some of the value of these kinds of orchestrated conversations is hopefully evident. For a start, they both enrich the quality agenda because they import people’s ‘lifeworlds’ into healthcare settings—providing a platform for lived experiences and ensuring that they are taken seriously. EBCD places some emphasis on making services more responsive to the experiences of patients and families but it does so by helping to build relationships with staff and by encouraging mutuality and dialogue. Schwartz Rounds place an emphasis on staff experiences and well-being but meetings will often engage with patient perspectives and be centred around patient voice. These kinds of exchanges will clearly also—indeed are meant to—bring different perspectives to the surface including more or less manageable tensions.

Opening up these kinds of conversations involves lowering some of the protective interpersonal and intrapersonal boundaries that are often in place when people are fulfilling a professional role or maintaining a public persona. In turn, this involves shifts in the focus of communication and shifts in style and register. Expert forms of discourse—for example, about the evidence-base, protocols or care pathways—remain relevant but are manifestly insufficient. Part of the purpose of these examples of orchestrated conversations is to help reconnect participants more directly with their own humanity and that of others. This requires different registers of communication, enabling engagement with practical and ethical uncertainty, and both mitigated and unmitigated suffering—the hard to fathom and the hard to bear. Such conversations hold together a range of concerns and possible contestations but, at the same time, they provide opportunities for joint working and for strengthening relationships—potentially sustaining motivation and forging solidarity.

This summary account indicates some of the work that is achieved through orchestration processes. Some analogous conversations might happen without orchestration but these will be much more dependent on pre-existing relationships, individual choices and virtues and on felicitous circumstances. Where they do occur they will also tend to be much more vulnerable to being foreshortened or cramped by the real or perceived power hierarchies between participants. Nonetheless, we do not wish to single out or privilege orchestrated conversations and in the next section we will turn our attention to the value of more routine conversational exchanges.

The potential contribution of conversations to healthcare quality improvement is, we hope, already emerging—horizons can be extended, and divergences, tensions and uncertainties can be brought to the surface. A careful and thoughtful evaluation of Schwartz Rounds in England describes the rounds as ‘countercultural’ (Maben et al. 2018). Part of what is meant here has been indicated in our discussion of the change in style and register that they entail. But their countercultural nature is chiefly explained in the evaluation report through a contrast with the prevailing emphasis in healthcare on problem solving, and associated with that, on comparative urgency. Of course, this emphasis makes sense—healthcare institutions do not exist as a collection of academic seminars. On the other hand, it is obviously important that there is some check on pragmatism and immediacy, and Schwartz Rounds are an example of a different orientation to time—which is why the same evaluation study characterises them as a ‘slow intervention’ (Maben et al. 2018). (Many similar things could also be said of EBCD because this too involves ‘standing back from’ and ‘interrupting’ the demands of ‘delivering a service’ in order to support long-term thinking and activity centred around reconsidering its purpose and configuration.)

Later, we will say more about how conversations might be seen as a response to normative complexity. But we can perhaps begin by noting that we are not suggesting that only wholly ‘open-ended’ conversations are suited to dealing with normative complexity. Rather, we would argue that both more and less directed conversational exchanges have important, and arguably complementary, roles. Normative complexity poses a challenge to the decision-making inherent in practical problem solving, but it does not ‘cancel out’ the possibility of defensible decision-making. At the same time, it is important to acknowledge that decision-making is sometimes very difficult and can require managing dilemmas, at least parts of which remain unresolved and accompanied by uncertainty, untidiness and practical and ethical ‘loose ends’.

## Learning from everyday conversations: ‘small talk’ and ‘catching up’

In this section, we will briefly consider some of the many conversations that are contained within routine healthcare encounters. This is clearly a huge topic which we cannot treat adequately here. By way of an example, we will confine ourselves to reflecting on a few issues associated with support being provided in patients’ homes. We choose this area in part because it continues one of the themes developed above—the business of crossing, and communicating across, potential boundaries—those between professional and personal spheres and between biomedical and ‘social’ domains.

The label conversation does not stick easily onto routine healthcare encounters: it seems to be somewhat too slippery. Given that healthcare practices are both heavily professionalised and often decision-oriented or action-oriented, there is usually some formal description of particular exchanges: they are consultations, history taking, shared decision-making and so on. While such exchanges often include conversational components as important elements something more is going on in them than ‘just’ conversation and, at the same time, the idea of a conversation seems itself to point to something beyond the more official description—perhaps something ‘surplus’ and potentially unruly (Hudak and Maynard 2011; Nordfalk et al. 2019).

This is one of the things illuminated by Macdonald’s analysis of the work of homecare nurses, which is presented as a contribution to understanding ‘the unrecognised but powerful nature of conversations between nurse and patient, which lie at the heart of nursing practice’ (Macdonald (2016, 1)). Macdonald focuses on the ‘small talk’ that goes on between nurses and patients during home visits. Such visits involve attending to some immediate health-related problem—what is described as their ‘transactional purpose’ but they also have a more ‘relational’ character (Cheepen 2000; McCarthy 2000).

In some ways, ‘small talk’ is simply a social necessity. In very many situations, but especially in spending time with people in their homes, conversational interaction is a strong social norm and its absence, even if feasible, would simply be rude and signal a disrespectful and uncaring attitude. However, Macdonald sees conversations as playing an important role for these nurses over and above fulfilling a general social norm. Indeed, for that reason she is reluctant to define ‘small talk’ in terms that are sometimes associated with it—as being trivial or peripheral. Rather she adopts an account of ‘small talk’ as ‘off-topic chat’, that is, chat which is ‘not concerned with the explicit purpose for which the speakers are together’—here, presumably, the primary clinical or ‘transactional’ purpose (Macdonald 2016, 3 based on Coupland 2000).

The nurses that Macdonald is studying work under considerable time pressures and they are often limited to fairly brief conversational exchanges and yet these sometimes fragmentary conversations can be seen as accomplishing a great deal and as ‘pivotal’ rather than marginal: they help to build and maintain a relationship, including sometimes a sense of continuity; they allow for the navigation and diplomatic handling of sensitive tasks and topics; they enable the nurses to acknowledge (and hence to some degree to share) the emotional burden of ill-health and they provide a foundation for ongoing partnership working between nurses and patients.

One reading of this shift in emphasis from the transactional to the relational, however, might be somewhat sceptical. It is possible to conceive of the relational here in heavily instrumental terms—as merely the transactional by other means. To the extent that small talk is orchestrated and directed towards specific transactional ends by the professional then it changes little except the feel of the encounter. Indeed, if and where we see ‘small talk’ simply as a mechanism for getting the (same) job done then we might also want to call into question the idea that it is actually that ‘off-topic’. However, Macdonald’s account also points us towards some more radical and disruptive possibilities. In entering into relationships, nurses are opening up new possibilities which include them, to some degree, listening to and being responsive to the concerns of patients. This can sensitise the nurses to patients’ perspectives and help them see specific health problems in a broader context, including redirecting attention to other needs and concerns that can to some degree be responded to (eg, through small interventions or by helping with access to other agencies, etc). At the least, this can help ensure that nursing responses are more personalised. However, this is not just about ‘adding in’ a degree of more social or ‘holistic’ awareness. Macdonald echoes Gawande’s recognition (Gawande 2014)—reflecting on observations of the conversations of palliative care nurses on homecare visits—that such conversations can ‘dramatically redirect clinical effort in a more helpful way for patients’ (Macdonald 2016, 3). Once again this suggests that a neat distinction between the instrumental and non-instrumental is unhelpful—in shifting away from a narrow predefined ‘task-completing’ conception of instrumentalism towards more open-ended forms of relating, then agendas are enlarged *and* practical ends are achieved.

Because ‘small talk’ can, among other things, enable health professionals to re-experience and rethink what they are doing, reflecting on the role and generative potential of conversations can become an important source of learning. Indeed, this is the explicit rationale that Macdonald offers for her work. There are other important practical and policy-related implications of this kind of analysis. What if the time and space for conversations becomes squeezed or even erased? Macdonald raises this worry both in relation to the increasing emphasis on technological solutions to ‘transactional’ healthcare and in relation to the steadily rising intensification of the working schedule of, and expectations on, nurses. This worry also features prominently in related scholarship on nursing work in people’s homes described by Adams et al. as involving ‘inherently complex relational care work that is often invisible in target-driven service cultures’ (Adam, Robert, and Maben 2013, 468).

The study by Adams et al. looks at the routine conversations that take place between community nurses who provide home care for patients. They borrow the shorthand, ‘catching up’ from the nurses themselves who use it to capture that feature of their working life and practices that involve a chance to interact with one another. These ‘catch ups’ provide a series of opportunities to informally update one another on what they have done and some of the concerns they have experienced that day, or since the last catch up. However, as with ‘small talk’ with patients these exchanges between healthcare professionals are not simply ‘transactional’. They are an expression of collegiality and, in many cases, of friendships that extend beyond the job and which include, for example, the exchanging of cards and gifts. By comparing a service where ‘catching up’ is relatively flourishing and one in which it has been put under severe strain by ‘throughput’ defined performance measure and ‘efficiency savings’, the study shows how both the quality of life of nurses and the quality of service provision are underpinned by these conversational exchanges, and how intimately related these two considerations are.

Nurses see these informal working relationships and enactments of professional collegiality as sustaining and as central to their well-being. Some of them describe this in plain terms, for example, they ‘“remind you that you are valued and cared for”, “make you feel that you want to come to work”, “keep you going” and “stop you feeling isolated” and “are the only reason I stay here”’ (Adam, Robert, and Maben 2013, 427). ‘Catching up’ also directly informs the care they can offer, and the authors use case studies to illustrate how the chance to hear stories about, and compare notes about, patients and families shapes their ability to understand and deal with sometimes very demanding situations and caring relationships. In the absence of ‘catching up’, the case studies show that the quality of care can suffer and, on occasions, be reduced to ‘delivering’ the minimum transactional tasks.

Overall, the authors highlight ‘the distinctive value of informal workplace “catch ups” for nurses to manage the inherent challenges of good home care for patients and to develop a shared ethic of care and professional identity….’ (422). ‘‘‘Catching up” often drew staff into conversations about the experience of giving relational care to patients and families. These conversations also involved staff giving advice, support and relational care to one another’ (426). In other words, this work reinforces the importance of conversations in personal and professional formation—in supporting reflective practice, enabling and embodying collegiality and informing the management of complex cases. As discussed in the last section, conversations do not simply provide a commentary on practice, they can also constitute new practices by building different kinds of relationships.

It is notable that there is something about the ‘superfluity’ of conversation—at least as compared with a narrow conception of purpose—that helps unlock exchanges and insights that can become straightforwardly useful in improving care-giving. The sense of perspective that comes from being enabled to suspend a narrowly task-oriented mindset, and to be able to relate to others in more responsive ways is what enables a changed re-conceptualisation of, and re-engagement with, nursing practice. Yet, it is precisely this kind of perspective that can be damaged by the imperative for efficiency, where this is narrowly construed. The threat that is posed to everyday conversations by resource constraints and, in particular, by short-sighted managerial pressures (Sujan, Spurgeon, and Cooke 2015), is very serious. It is illustrative of, and helps to explain, the constant risk of ‘aridity’ discussed in the last section. Orchestrated conversations seem to become all the more necessary, and all the more ‘countercultural’, when the well of routine conversation runs low.

The discussion of ‘small talk’ and ‘catching up’ for those working across the boundaries of healthcare institutions and homes echoes many of the themes that were signalled in the previous section. There is the simple burden of ‘carrying on’—coming to terms with what is feasible—and there are puzzles about whose agendas matter, and what to do when nurses and patients may want to aim in different directions, including the balance between sustaining relationships on the one hand and a degree of ‘constructive conflict’ on the other (Entwistle et al. 2018).

## Bringing multiple perspectives to quality

Up until now, we have been investigating the value of conversations through some practical examples. Now, we want to specify a feature of conversational exchanges that we suggest is particularly important for enriching quality discourses—namely the way that conversations rest on the recognition of multiple perspectives and do not necessitate the elimination of perspective. By way of a somewhat crude contrast we can imagine an approach that aims for a completely unified account of quality—for example, and using a deliberately simplified model, we can imagine a manager in a particular setting who wishes to provide ‘good care’; he or she recognises that this involves people doing a ‘good job’, patients having ‘good experiences’ and the care having ‘good outcomes’. This obviously involves engaging with a range of people, collecting insights and data and combining them together into a single picture. Up to a point, this seems like a sensible (and recognisable) model—it is certainly much better than not working with multiple perspectives at all. But, we are suggesting, it is important to note that while something is achieved in the production of a unified account there is a risk that much else is lost. To approach this topic, we will take a turn in a slightly more abstract direction.

We have thus far relied on a ‘common sense’ account of conversations and looked at literal conversations that take place in meeting spaces including people’s homes. But there are also, of course, more extended, elaborated and metaphorical uses of the term. One of the most famous is to be found in Michael Oakeshott’s account of ‘The Conversation of Mankind’, as part of his critique of rationalism and reductionism. Oakeshott sees it as important to distinguish between different ways of experiencing the world—different ‘modes’—so that we avoid the mistake of supposing that all of our thought and activity is of one sort and can somehow be combined into one commensurable currency. There are different kinds of understanding and ways of proceeding and there is not one version of ‘rationality’ that runs through them. In talking about ‘modes’, Oakeshott has in mind huge spheres such as ‘science’, ‘history’ and ‘poetry’, and yet it is not difficult to see how analogous kinds of modal differences arise in healthcare where, for example, biomedical, biographical and social perspectives all have relevance but do not necessarily add up to an integrated whole. Different methods, approaches or forms of reasoning are good for different purposes but do not stretch across everything. It is against this background that Oakeshott invokes the importance of conversation. Separate spheres and perspectives do not have to be seen as completely cut-off from one another because they can be brought together in a conversation between ‘voices’—here ‘voices’ refers to modes but we can also imagine voices being represented by different people or coexisting within a single person. Oakeshott’s conception of a conversation is in one sense very expansive but it places a heavy stress on the way in which conversation can open things up rather than closing them down, by linking and retaining diverse perspectives rather than reconciling or dissolving them:

In a conversation the participants are not engaged in an inquiry or a debate; there is no 'truth' to be discovered, no proposition to be proved, no conclusion sought. They are not concerned to inform, to persuade, or to refute one another, and therefore the cogency of their utterances does not depend upon their all speaking in the same idiom; they may differ without disagreeing. Of course, a conversation may have passages of argument and a speaker is not forbidden to be demonstrative; but reasoning is neither sovereign nor alone, and the conversation itself does not compose an argument… Conversation is not an enterprise designed to yield an extrinsic profit, a contest where a winner gets a prize, nor is it an activity of exegesis; it is an unrehearsed intellectual adventure. It is with conversation as with gambling, its significance lies neither in winning nor in losing, but in wagering. Properly speaking, it is impossible in the absence of a diversity of voices: in it different universes of discourse meet, acknowledge each other and enjoy an oblique relationship which neither requires nor forecasts their being assimilated to one another. (Oakeshott 1962, 197)

Although Oakeshott is essentially a conservative thinker, perhaps even someone invoking a premodern sensibility, there are clear parallels with familiar postmodern themes here—a suspicion of appeals to a singular ‘reason’, scepticism about ‘meta-narratives’ that capture everything and an acceptance of pluralism that includes recognising that we operate from within multiple discourses rather than from above them. Oakeshott’s high-level and ‘evaluative’ vision of conversation also resonates, although on a much grander scale, with the observations made earlier in the paper that the language of conversation is unsuited to strongly directed activities. While communicative exchanges can be harnessed to making decisions and achieving institutional or professional goals, when we describe aspects of such exchanges as conversations we are also invoking something that ‘exceeds’ the instrumental and reaches beyond the specific activities in question. A particular issue may be resolved or a problem solved but this does not erase the possibility of ongoing conversation or the space between perspectives.

Part of what is contained in this account is that conversations are being treated as an example of what Habermas calls ‘communicative’ rather than ‘strategic’ action (Habermas 1987). They provide a basis for ‘mutual understanding’, which involves attention to, and a genuine interest in grasping, what matters from the standpoint and experience of others, rather than the adopting of a similar seeming interest in order to further one’s own agenda. Here, ‘mutual understanding’ is contrasted with one party dominating or using the other party—echoing the distinction that Oakeshott makes between conversation and ‘winning and losing’. In principle, this distinction offers a useful heuristic, but in practice, as we saw in the previous section, there is no easy line to draw, or perfect insulation between, the relational and transactional dimensions of encounters. An interest in understanding and mutuality is important for its own sake and it may separately turn out to be practically useful because understanding can reorient action. Acknowledging this ‘double value’ is not at all to accept that conversations tend to collapse back into ‘strategic action’, but it does require us to note the prevalence of power differentials between different parties and the risk of such collapse happening sometimes.

Standish, reflecting on Oakeshott’s conception of a conversation, argues that conversation depends on a degree of social coordination but need not require ‘cooperation’ in the restricted liberal sense—that is conversation does not require an agreed and shared project or purpose, or the assumption of a ‘neutral field’ designed to support participants in the pursuit of their separate projects. Rather conversation reflects and sustains some more minimal notions of respect and mutuality, recognising the possibility of engagement across difference. While conversation allows for people’s perspectives and trajectories to remain ‘intact’, they may well not do so:

Conversation is then the field in which I might discover what my projects might be.

… There is here the suggestion of a turning of thought such that it cannot proceed solely, and in many respects does not succeed best, when it travels along straight, systematic lines: openness to conversation, a readiness to be turned (to be shaped, fashioned, sometimes diverted, sometimes rebuffed), requires that I do not seek to shore up my own identity but rather am ready for new possibilities—that is, ready to become. (Standish 2016, 122)

Once again, we can connect these abstracted discussions of (perhaps idealised) conversations with the specially orchestrated and more routine conversations considered in the previous sections. A conversation is not the same as a consolidation. It can provide an arena for diversity and represent multiple vantage points that may be sustained and interact within it in dynamic ways. But it is, by the same token and as Standish indicates, an opportunity for personal or professional formation and adaptation and for practical change.

Returning to the simplified model with which we began this section—a manager ‘adding up’ perspectives to obtain a quality ‘sum total’—we are simply stressing that when there are multiple perspectives in play there is no reason to suppose that these can be wholly and neatly unified. Although for specific purposes there may be some value in pressing assorted jigsaw pieces into a single picture, it is equally important to see these pieces in the context of the pictures they originate from. For example, thinking about doing a ‘good job’ involves something different from, and more than, thinking about ‘good experiences’ and vice versa. What counts as ‘good outcomes’ may be something that is contested both within and across professional groups and patient groups (Owens et al. 2017). Furthermore, it is possible to shift focus between both actors and settings. Home provides one setting that parents and carers ‘manage’ and in which they may strive to do a ‘good job’—even including the application of ‘clinical know how’ more normally associated with professional expertise. Equally, as is now widely recognised, it is also important to address ‘good experiences’ for staff, what this means, and how that contributes towards, or may support them in doing a ‘good job’ (Hall et al. 2016; Maben et al. 2012). Thinking about healthcare quality involves looking across many such intersecting axes. In each case there are not only multiple actors but also large and diverse theoretical and empirical literatures—whether about the nature of expertise, the costs and benefits of standardisation, the understanding and measurement of well-being, the social determinants of resilience and so on—all of which are relevant but none of which is sufficiently rich on their own to define quality. Producing an account of healthcare quality that is rich enough to be plausible involves seeing each setting, each group of actors and each set of axes and related literatures, as being made up of a network of interlinked conversations.

## Conversations and the challenge of complexity

Before trying to pull some threads together, we will say more about the nature and significance of normative complexity and begin to sketch out how conversations can help manage the normative complexity of healthcare quality. We have suggested that cutting across the causal complexity produced by a multiplicity of actors and relatively open and interlinking subsystems is the additional complexity produced by diverse conceptions of what matters. There is a need to be cautious both about what will happen and about what we should take to be valuable. This combination intensifies the already considerable difficulty of making judgements about improvement.

The literature on complex systems shows that in planning or evaluating interventions we need to be conscious of the way that ‘feedback loops’ can interrupt or reinforce what is intended (with in each case the potential for both welcome and unwelcome side-effects). What we have labelled as normative complexity is different from but can, for the purpose of this argument, be understood in key respects as broadly analogous to explanatory complexity (Davidoff 2019; Braithwaite, Churruca, and Ellis 2017). Just as it is impossible to predict what might happen by focusing in on simple singular causal chains because of the way diverse actions, subsystems and environments interact, it is also impossible to determine what counts as a success without engaging with value diversity and the complex interactions between constellations of values. In both cases, the challenge is not simply that we need to do some more ‘complicated calculations’—as if ‘robust prediction’ or ‘objective evaluation’ depended on examining more variables in more sophisticated ways. Rather the kinds of complexity in question mean that there are arguably inherent limits to how far it makes sense to aspire to resolve uncertainties—we may simply be looking to rule out a variety of bad answers in order to leave a range of best judgements plus caveats. This deep-seated kind of uncertainty is well understood in relation to explanatory complexity but its relevance to normative complexity is less often addressed.

First, there are a large range of valued things that might be relevant to quality—including the Institute of Medicine’s ‘six dimensions’ of quality (effectiveness, safety, patient-centredness, timeliness, equity, efficiency) and other factors that are widely accepted to be important but often get less official recognition such as staff well-being, environmental sustainability, cultural responsiveness and so on (Institute of Medicine 2001). Second, there are different stakeholders who will often operate with different constructions of, and weightings for, the range of relevant values. Third, there is plenty of scope for disagreement about roles and obligations—who might reasonably be expected to do or prioritise what in order to make things ‘better’? These different axes of normative complexity interact with each other in multiple ways (and, as we will come to in a moment, with explanatory complexity). Values cannot be neatly separated out but rather inform, shape and co-constitute one another (adding another axis to feedback loops). We should arguably hesitate to talk about efficiency without thinking about staff well-being; to make claims about effectiveness without being mindful of sustainability; to pursue person-centredness without paying attention to recognitional aspects of justice or indeed to invoke any specific value dimension without an awareness of the broader constellations in which it is embedded. Given the range of contestations about what each of these values means, how they can be prioritised and combined and who should do what, then determinations of success become challenging and volatile.

In practical decision-making, normative complexity and explanatory complexity overlap. Improvement interventions are based on a set of value assumptions, for example, assumptions about what combination of processes and outcomes are valuable; which of these considerations matter more or less and who has what kinds of roles and obligations. Even quite small causal or normative ‘feedback loops’ mean that such assumptions may quickly be judged to be mistaken, or—because of the feedback—actively become misplaced, with the result that good intentions result in bad effects of various kinds and degrees. This is a pervasive phenomenon but one area where it has become regularly ‘named’ and recognised as a danger in health systems is sometimes labelled as ‘Goodhart’s law’—that is, when promising measures are made into targets, or simply given salience in the system they are measuring, they tend to lose their promise (Strathearn 1997). This is because directing institutional attention towards something changes the way people interpret their responsibilities. Someone introducing a specific intervention with a particular desired effect in mind, for example, increasing measured compliance with a safety standard, needs to be aware that it may produce other effects as well as, or even cancelling out, the desired effect. It may, for example, produce forms of ‘surface compliance’ (eg, compliance on paper) which to some degree distorts data and obscures vision, or it may lead to a re-prioritisation of effort with risks and costs elsewhere. Even from this simple case, it is clear that making a judgement about whether an intervention can be seen as an improvement is inherently complicated and unstable such that it may be reasonably contested by different actors. This is because even when we feel reasonably confident we can trace the effects of interventions, it may be difficult to work out how to balance the value of the intended and unintended effects. In other words, when deciding what to do we need to take into account both explanatory and normative complexity.

Conversations arguably have a particularly important role within organisations as and when normative complexity becomes recognised. For example, Bushe and Marshak argue that what they call ‘Dialogical' Organisational Development (OD) is particularly suited to those situations in which organisations do not know how to go on—because they are trapped in repeating interventions that have been shown to be ineffective or because they are dealing with ‘wicked problems’ where there is little agreement about what is happening or what would count as a solution (Bushe and Marshak 2016). Dialogical OD is presented as an alternative to a more strategic ‘problem-solving’ paradigm which starts with a vision of the desired outcomes and then develops and implements an action plan. By contrast, ‘Dialogical approaches work by fostering generativity to develop new possibilities rather than problem solving, altering the prevailing narratives and stories that limit new thinking, and working with the self-organising, emergent properties of complex systems’ (Bushe and Marshak 2016, 407).

Bushe and Marshak explicitly cite ‘conversations as one of eight ‘key premises’ for Dialogical OD:

The social construction of reality occurs through the conversations people have, everyday. Change requires changing the conversations that normally take place. This can occur from changing who is in conversation with whom (eg, increasing diversity, including marginalized voices), what is being talked about, how those conversations take place, increasing conversational skills, and by asking what is being created from the content and process of current conversations. (408)

What is being highlighted here is the role of conversations, and the adaptations and improvisations they contribute towards, in managing feedback loops. That is, as change emerges we can respond from within the midst of it, proceeding iteratively and refining, correcting or repairing developments in more fine-grained and flexible ways than is enabled by top-down strategic approaches.

There are some striking parallels between these insights from Dialogical OD and the lessons for dealing with complex healthcare systems that Braithwaite offers for quality improvement more broadly. For example, Braithwaite stresses the importance of the ‘micro’ and ‘granular’ in adaptation, and the need to make use of the ‘informal system’ and not just the ‘formal system’ of healthcare, taking ‘every opportunity to bolster communication, trust and interpersonal relations’ (Braithwaite 2018).

We are suggesting that conversations have a very important role to play in the response to complexity. Both the substance and the process of conversation are relevant. Those who are trying to steer their way towards healthcare improvements may usefully debate knotty causal and normative questions. This means asking “do we understand what is happening here?” and “do we know how to attach value to it?” To some extent organisations, in order to function, are required to come to (provisional) conclusions about these matters but this is quite consistent with ongoing differences of interpretation and emphases between different parties. People representing different professional or patient communities may have good reason to disagree about whether or not things are getting better in ways that they judge to be important. An organisation that is interested in quality will be interested in coming to conclusions and equally interested in keeping alive debates and the multiple perspectives they reflect, and in building and maintaining relationships between different constituencies.

## Cultivating conversations

We hope to have indicated a range of ways in which conversations can contribute to healthcare quality improvement, and in particular their value in handling normative complexity. In this final section, we will summarise where we think we have got to and also indicate some practical implications.

The conversations we have discussed are more or less mundane, and range from being about immediate concrete things to relatively abstract concerns. The everyday conversations that occur within the context of healthcare have the potential to both embody and inform better care. They contribute to relationship building and help uncover the perceptions, wants and needs of a range of actors, expanding the range of considerations judged relevant to decisions and actions. Orchestrated conversations (such as those within EBCD and Schwartz Rounds) that are explicitly intended to support healthcare improvement or underpin quality can help crystallise and amplify many of the complexities that are managed within routine conversations, can build bridges between diverse voices and perspectives, and can thereby help to extend the ‘quality agenda’. All of these conversations thus contribute to information flows in healthcare, facilitate ongoing micro-accommodations between actors and indicate areas where more far-reaching re-design or reform may be needed. There is also an analogous role for conversations at a more abstract level, for example, between different currents of professional reflexivity and scholarship. These latter conversations allow us to approach healthcare quality with the full range of theoretical as well as social resources (Cribb 2018).

Conversations, we have suggested, can help address normative complexity in two ways, roughly corresponding to the distinction sometimes made between ‘engineering’ and ‘enlightenment’ approaches to change (Davies, Nutley, and Smith 2000). First, they can increase the chance that the full range of relevant voices and values have been considered. Of course, this is nothing like as strong a claim as arguing that they increase the chance of the multiple, and sometimes competing, values at stake being weighed and combined in relatively defensible, let alone the best possible, ways. (To make the latter claim raises practical and ethical challenges including, eg, about the background communicative conditions (Habermas 1987). Second, conversations can contribute to improvement in more diffuse ‘context-strengthening’ ways both by forging links between diverse voices and by increasing awareness of normative complexity—about the breadth of potentially relevant values and the resulting ambivalences, tensions, dilemmas and uncertainties. It is worth stressing that ‘intervention-informing’ and ‘context-strengthening’ contributions are not mutually exclusive. The bigger and broader the literacy about normative complexity, the greater the chance of specific interventions being intelligently informed. Yet however carefully we try to address normative complexity in the design and conduct of interventions, we are also bound to be left with remaining contestation, untidiness and loose ends. Ongoing attention to this contributes to improvement in a context-strengthening ‘enlightenment’ way. The examples we cited earlier roughly map onto this distinction: with EBCD and ‘small talk’ sitting closer to the ‘intervention’ model and Schwartz Rounds and ‘catching up’ sitting closer to the ‘context-strengthening’ model.

The ‘enlightenment’ ways of talking about the potential value of conversations arguably have much stronger resonance with the way people characterise the contributions of arts and humanities to healthcare rather than the more technicist framings commonly found in clinical science and sometimes in health services research. This is unsurprising because the latter often work with a background notion of ‘consolidating’ knowledge, as discussed above in the contrast we drew between consolidation and conversation. Here, the danger is that the perspectives of patients or other actors risk being treated merely as data to be harvested in the building of better—often meaning simpler and more unified—models. If, as we have been arguing, one of the characteristics of conversations is that they leave room for a plurality of perspectives and stories, and can ‘contain’ complexity, including contestations and loose ends, then they might be seen as more prosaic ‘cousins’ to art forms such as literature.

Indeed, there are strong resonances between the rationale offered for the cultivation of a ‘slow’ context-strengthening activity such as Schwartz Rounds and the kinds of justification that are regularly cited for studying medical humanities (Kollmer-Horton 2019). More specifically, Schwartz Rounds have been evaluated as increasing staff’s ‘empathy and compassion for colleagues and patients’ (Maben et al. 2018). This echoes Nussbaum’s influential analysis of literary fiction, which centred on its capacity to foster empathy (Nussbaum 1992). However partial, this correspondence suggests that one of the key components of context-strengthening work may be the cultivation of character including moral imagination. Personal and professional formation arguably provides one important element of the contribution of conversations to healthcare quality climates and practices. But, of course, formation is not something to be seen in purely individualistic terms; rather, we need to address the social aspects of, and conditions for, both personal and professional formation.

As we have noted healthcare landscapes can sometimes be arid. While there can be immense value in creating ‘oases’ for rich conversations, these arguably should not be oases at all. We might well ask how to make such conversations more commonplace. This takes us close to the concerns discussed in the last section in relation to the idea of Dialogical OD. In the terms just introduced, we can ask about how to ‘engineer’ the possibilities of ‘enlightenment’. Here we think it is worth following the lead of Kerasidou et al. who suggest that the focus of questions about empathy could usefully switch from individuals to systems (Kerasidou, Baeroe, and Berger 2020). They define empathetic systems or institutions as ‘systems and institutions that are structured and organised in such way as to create conditions that facilitate empathetic interactions in a non-arbitrary way throughout the whole service’ (*page numbers unavailable at time of submission*).

They also begin to sketch out some of the characteristics of empathetic systems. For example:

A healthcare system that aims to provide adequate and empathic care, must therefore be self-reflexive regarding its own conditions of power.

…… Rethinking the indicators used to assess quality of care might be one way to achieve this.

We suggest that this same project should be extended. In addition to the conditions for empathy, we should ask about the conditions for creating structures and cultures that enable constructive engagement with normative complexity. This includes work on the conditions for supporting moral imagination. What are the characteristics of a system or institution that recognises, values and harnesses different perspectives on how to conceptualise and pursue quality? This is a huge question and one that could require a substantial programme of work. But we are tempted by the provisional thought that one sign of a ‘morally imaginative setting’ might be that it was hospitable to conversations about quality that open up, and seek to address, the normative complexity of healthcare.

## Data Availability

No data are available. No data used in this study.
